# Scarlet Fever in One Family

**Published:** 1883-03

**Authors:** Addison H. Foster

**Affiliations:** 779 W. Monroe Street


### ARTICLE III.

### SCARLET FEVER IN ONE FAMILY. By ADDISON H. FOSTER, M.D.

The last of September, 1882, a child A, aged 11, had a mild throat attack with some fever, so slight I was not called.

October 11, child B, aged 6, became feverish with sore throat and furred tongue. On the 13th, a scattered, coarse and slightly papular eruption appeared, and by the 15th a well-marked, welldiffused characteristic scarlet fever rash was developed, and remained a full week, with high fever and coated tonsils.

October 15, child C, aged 9, became feverish, with furred tongue and cankered tonsils, and was quite sick for three days, without any eruption whatever.

October 22, child D, aged 2, was taken like child C, and was sick for three days without any eruption.

November 6, or fifteen days later, child E, aged 4, was taken like the two preceding, quite as severely for three days, but with no eruption, and six days later, November 12, child F, aged 13, had a similar attack, but milder, with no eruption.

Seven days later, on November 19, or thirty-seven days after his first attack, child B had a second attack with less fever and sore throat, but with as well-defined and general scarlet fever eruption as before, which remained about five days.

Twelve days later, on December 1, the child C, aged 9, was taken with fever, sore throat, and well-marked scarlet fever rash, and was sick in bed for a week.

Fourteen days later, on December 15, the child D, aged 2, became sick for a week with fever, sore throat, and the same kind of eruption.

Thirteen days later, December 27, the child E, aged 4, was taken like the rest, and was in bed for a week with fever, throat trouble and eruptions.

Child F, aged 13, had no second attack.

Child A had a peculiar granular-looking surface upon the swollen tonsils and borders of the palate ever after the first attack until about two months after the second, which finally yielded to direct daily application of tincture of iodine and glycerine, equal parts.

None of the dreaded sequelæ occurred in any of the cases.

The case of the first child I believe to be of specific scarlet fever poison, contracted at school, as child B was not in school. And in this case, as in many other I have seen, the younger ones at home, the least exposed, were the first to develop the disease in all its characteristics.

It is not usual to see a large family come down singly, and at such long intervals. Although we meet variable periods of incubation in the same household, the disease occurs more frequently in groups, as though two or three had been infected at the same time.

The striking feature of this history is the recurrence of sickness in the same order of individuals as at first; and, that the child A should so soon repeat his eruptions, six months being the quickest repeat in my previous experience.

779 W. Monroe Street.

